# A regression method for EEG-based cross-dataset fatigue detection

**DOI:** 10.3389/fphys.2023.1196919

**Published:** 2023-05-30

**Authors:** Duanyang Yuan, Jingwei Yue, Xuefeng Xiong, Yibi Jiang, Peng Zan, Chunyong Li

**Affiliations:** ^1^ Shanghai Key Laboratory of Power Station Automation, School of Mechatronics Engineering and Automation, Shanghai University, Shanghai, China; ^2^ Beijing Institute of Radiation Medicine, Academy of Military Medical Sciences (AMMS), Beijing, China

**Keywords:** fatigue detection, cross-dataset, EEG, regression method, self-supervised learning

## Abstract

**Introduction:** Fatigue is dangerous for certain jobs requiring continuous concentration. When faced with new datasets, the existing fatigue detection model needs a large amount of electroencephalogram (EEG) data for training, which is resource-consuming and impractical. Although the cross-dataset fatigue detection model does not need to be retrained, no one has studied this problem previously. Therefore, this study will focus on the design of the cross-dataset fatigue detection model.

**Methods:** This study proposes a regression method for EEG-based cross-dataset fatigue detection. This method is similar to self-supervised learning and can be divided into two steps: pre-training and the domain-specific adaptive step. To extract specific features for different datasets, a pretext task is proposed to distinguish data on different datasets in the pre-training step. Then, in the domain-specific adaptation stage, these specific features are projected into a shared subspace. Moreover, the maximum mean discrepancy (MMD) is exploited to continuously narrow the differences in the subspace so that an inherent connection can be built between datasets. In addition, the attention mechanism is introduced to extract continuous information on spatial features, and the gated recurrent unit (GRU) is used to capture time series information.

**Results:** The accuracy and root mean square error (RMSE) achieved by the proposed method are 59.10% and 0.27, respectively, which significantly outperforms state-of-the-art domain adaptation methods.

**Discussion:** In addition, this study discusses the effect of labeled samples. When the number of labeled samples is 10% of the total number, the accuracy of the proposed model can reach 66.21%. This study fills a vacancy in the field of fatigue detection. In addition, the EEG-based cross-dataset fatigue detection method can be used for reference by other EEG-based deep learning research practices.

## 1 Introduction

Fatigue is one of the major factors leading to human errors, which is accompanied by impaired attentional control, decreased individual alertness, and poor performance in tasks (Liu et al., 2020). These are dangerous for certain jobs requiring continuous concentration, such as pilots, vehicle drivers, and helmsmen (Liu W. et al., 2019). To avoid failures caused by fatigue, researchers are working on ways to detect/monitor fatigue using different types of signals. The first is based on individual behavior, including physiological responses, such as eyelid-related parameters (Hu and Zheng, 2009), facial expressions (Liu Y. et al., 2019), head movement (SMITH et al., 2016), percentage of eye closure (PERCLOS) (Zheng and Lu, 2017), and the performance observed during the execution of specific tasks, such as reaction time (RT) and response accuracy (Huang et al., 2016; Liu et al., 2020; Zeng et al., 2020). The second is based on psychological surveys, such as the Karolinska Sleepiness Scale, Stanford Sleepiness Scale, and Chalder Fatigue Scale (Foong et al., 2019; Qin et al., 2020; Krigolson et al., 2021; Zeng et al., 2021). The third is based on physiological signals, such as electroencephalogram (EEG) (Gao et al., 2019; Peng et al., 2021), electrooculogram (Bulling et al., 2011), electrocardiogram (Murugan et al., 2020), or a combination of signals (Qi et al., 2018; Du et al., 2022). Precisely, EEG measures the potential difference produced from the electrical signals generated by the synaptic excitation of neurons to the scalp, and it can directly reflect the activities of nerve cells in the brain (Kostas et al., 2021; Liqiang et al., 2022). Therefore, it is considered to be the most effective method to detect fatigue.

At present, the fatigue detection method of within-subject and cross-subject has achieved outstanding performance. For the within-subject fatigue recognition, Yang et al. (2021a) proposed a complex network (CN)-based broad learning system (CNBLS) to realize fatigue detection based on EEG. The classification accuracy of CNBLS was 99.58%. Wang H. et al. (2021) introduced a new attention-based multiscale convolutional neural network–dynamical graph convolutional network model for driving fatigue detection. The two-class accuracy was 95.65%. For cross-subject fatigue recognition, Zeng et al. (2020) used the InstanceEasyTL method to detect driver fatigue, and the two-class accuracy was 88.02%. Liu et al. (2020) proposed a transfer learning-based algorithm using maximum independence domain adaption (MIDA), and it achieved an accuracy of 73.01% with all 30 channels for the two-class mental fatigue recognition. Wei et al. (2018) developed a subject-transfer framework for obviating inter- and intra-subject variability in drowsiness detection, and this framework remarkably reduced the required calibration time for a new user. In addition, in the emotion recognition field, Iyer et al. (2023) proposed the ensemble learning-based EEG emotion recognition system, and the ensemble model outperforms the compared methodologies with 97.16% accuracy for EEG-based emotion recognition on the SEED dataset. In the sleep stage classification, Sharma et al. (2021) used a discrete wavelet transform and discrete entropy to analyze EEG signals, studied the wavelet sub-band of EEG sleep records and its performance based on wave dispersion entropy, and finally obtained EEG features suitable for sleep stage classification.

However, when faced with new users under different datasets, the model of within-subject and cross-subject fatigue detection still needs a large amount of EEG data for training. It has poor applicability. The cross-dataset fatigue detection model has a strong practical application value because it does not need to be retrained, and it can directly detect fatigue states of new datasets. In order to get a general cross-dataset fatigue detection model, this study considers different sets in BCIs. In other words, different datasets have different label spaces (He and Wu, 2020). For fatigue detection, this means that the subjects of different datasets perform different fatigue-induced tasks. Different tasks have different feature spaces. Thus, the very effort of selecting different features for different tasks is a critical challenge for cross-dataset fatigue detection. However, the within-subject and cross-subject fatigue detection models are difficult to generalize knowledge to new datasets because they suffer the drawbacks of fully supervised learning and large-scale labeled datasets for training (Ye et al., 2022), and the label work process is prone to human bias and may also result in ambiguous annotations. In particular, each dataset has multiple subjects, so cross-dataset fatigue detection is a multi-source to multi-target domain problem.

Up to now, the methods of fatigue detection are mainly judged from facial expressions, physiological signals, and questionnaire surveys, but it is difficult to have a general fatigue detection model to adapt to various fatigue-induced tasks. To the best of our knowledge, no one has studied this problem previously. However, the idea of a cross-dataset has gained widespread attention in other fields, such as emotion recognition, sleep staging, and personal identification. Ni et al. (2021) used a domain adaptation sparse representation classifier to minimize the data distribution difference between datasets and then classify emotions for EEG collected from different subjects, different periods, and different devices. Eldele et al. (2022) used the adversarial learning framework called ADAST for automatic sleep staging. The framework can tackle the domain shift problem in the unlabeled target domain, which is a limitation to domain adaptation in sleep staging. Kong et al. (2018) proposed a method for cross-dataset personal identification based on a brain network of EEG signals. The method used brain functional networks and linear discriminant analysis (LDA) to classify personal identification. As can be seen from the aforementioned fields, domain adaptation is one of the main methods to solve cross-dataset problems (Chen et al., 2021; Ding et al., 2021). In addition, the method proposed in this study can also be applied to these fields.

In real life, there is a coherent sequence of changes in EEG variables during the transition from normal driving, high mental workload, and eventual mental fatigue and drowsiness, so fatigue detection should be a regression problem. However, current fatigue evaluation methods are mostly classification methods, aiming to divide the brain states into two or more alert and fatigue states (Yang et al., 2021b). This is a simplified version of regression analysis. To develop a model that can adapt to different fatigue-induced tasks, this study focuses on the following four points to carry out the specific content of this study.

(a) A regression method for EEG-based cross-dataset fatigue detection is proposed to detect the fatigue states of the new datasets collected for different fatigue-induced tasks. The method includes two steps: pre-training and the domain-specific adaptive step. The purpose of pre-training is mainly to extract specific features for different datasets. The domain-specific adaptive step is mainly to align specific features extracted from the pre-training step and mine the internal relationship between features. To validate the proposed method, a large number of experiments were conducted to compare the proposed method with state-of-the-art domain adaptation methods.
(b) In the pre-training step, this study designs a pretext task to distinguish data from the source or target domains. In this way, the specific features of different fatigue-induced tasks can be obtained. The pre-training step includes a common feature extractor and a domain discriminator. In this study, we have performed a lot of experiments to verify that the accuracy of pre-training steps is better than that of no pre-training steps, which proves the contribution of pre-training.
(c) In the domain-specific adaptive step, this study proposes an EEG-based domain-adaptive fatigue detection network. In addition, it includes a domain-specific feature extractor, domain distribution alignment network, and regression multilayer perceptron. Maximum mean discrepancy (MMD) is used to optimize the network parameters in the domain-specific adaptive step, which can minimize differences between the source and target domains.
(d) The attention mechanism is introduced to extract continuous information on spatial features, and the gated recurrent unit (GRU) is introduced to capture information on time series. This study also conducts experiments to verify the effectiveness of the attention mechanism and GRU.

The rest of this paper is organized as follows: Section 2 describes the proposed method. Section 3 presents the experiment and results. Section 4 discusses the results, and Section 5 concludes the paper.

## 2 Materials and methods

### 2.1 Problem statement

It is difficult to obtain a general model that is suitable for datasets because of different tasks. Therefore, if the model trained by one dataset is applied to another dataset directly, it will lead to performance degradation. When faced with a new dataset, the conventional method needs to undergo the calibration process, that is, to collect lots of new labeled data and train a new model for these data (Wang Y. et al., 2021). This is time-consuming and not economical.

Suppose we have labeled EEG samples from one dataset $\left\{X_{s},Y_{s}\right\}={\left\{\left(x_{s_{i}},y_{s_{i}}\right)\right\}}_{i=1}^{N_{s}}$ , denoted as the multi-source domain $D_{s},$ and unlabeled EEG samples from the another dataset $\left\{X_{t}\right\}={\left\{x_{t_{j}}\right\}}_{j=1}^{N_{t}}$ , denoted as the multi-target domain $D_{t}$ , where $X_{s}\in R^{d\times N_{s}}$ , $X_{t}\in R^{d\times N_{t}}$ , $Y_{s}\in R^{C\times N_{s}}$ , $x_{s_{i}},x_{t_{j}}\in R^{d}$ , and $y_{s_{i}}\in R^{C}$ is a one-hot vector, d is the feature dimensionality, C is the fatigue index, and $N_{s}$ and $N_{t}$ are the number of samples in multi-source and multi-target domains, respectively. However, the marginal distributions and conditional distributions of the feature space, and the fatigue index space of both domains are different due to the domain shift: $P_{s}\left(X_{s}\right)\neq P_{t}\left(X_{t}\right)$ and $P_{s}\left(Y_{s}|X_{s}\right)\neq P_{t}\left(Y_{t}|X_{t}\right)$ . Domain adaptation solves this problem by mapping the multi-source and multi-target domains to a new space R and then minimizing the distance $D_{s-t}$ between the multi-source mapping distributions $R_{X_{s}}$ and multi-target mapping distributions $R_{X_{t}}$ .

### 2.2 Datasets

#### 2.2.1 SEED dataset

The data were collected by Zheng and Lu (2017). A total of 23 subjects participated in the experiments. The experimental data collection scenario was a virtual-reality-based simulated driving scene. A four-lane highway scene is shown on a large LCD screen in front of a real vehicle without the unnecessary engine and other components. The vehicle movements in the software application are controlled by the steering wheel and gas pedal. During the experiments, the subjects were asked to drive the car using the steering wheel and gas pedal, and the scenes were simultaneously updated according to the participants’ operations. The 12-channel EEG signals from the hindbrain (CP1, CPZ, CP2, P1, PZ, P2, PO3, POZ, PO4, O1, OZ, and O2) and 6-channel EEG signals from the temporal lobe (FT7, FT8, T7, T8, TP7, and TP8) were recorded. The author of the dataset used independent component analysis filtering to remove noise, such as the artifact of eye movement, electromyography, and baseline drift. In this study, we filtered the dataset with 1-Hz high-pass and 50-Hz low-pass finite impulse response (FIR) filters. The processed data were finally downsampled to 128 Hz. The vigilance annotation method of the dataset used PERCLOS, which refers to the percentage of eye closure. Specifically, eye movements were simultaneously recorded using SMI ETG eye tracking glasses.

Data labels were defined in a way that classifies EEG data into three fatigue states (awake, fatigue, and drowsy) with two thresholds (0.35 and 0.7) based on the PERCLOS index. In addition, in the following study, this study uses “SEED_0” for awake, “SEED_1” for fatigue, and “SEED_2” for drowsy in the SEED dataset.

#### 2.2.2 Multi-channel dataset

The dataset consists of EEG signals collected by Cao et al. (2019). In the experiment, 27 participants were invited to the experiment. Fatigue and drowsy states were induced by a 90-min sustained-attention night-time driving task in an immersive driving simulator. The participants were tasked to drive and maintain the car in the center of the lane. Lane-departure events were randomly induced, which made the car drift to the left or right from the lane, and participants were asked to move back as quickly as possible by steering the wheel. In addition, their reactions were timed. The vigilance annotation method of the dataset used the RT, which provides a gauge of the subjects’ fatigue level. The preprocessed version of the dataset was used in this study. As described by the authors, the raw EEG signals were filtered by 1-Hz high-pass and 50-Hz low-pass FIR filters. Apparent eye blinks that contaminate the EEG signals were manually removed through visual inspection by the authors of the dataset. Ocular and muscular artifacts were removed by the automatic artifact removal plug-in of EEGLAB. The processed data were finally downsampled to 128 Hz.

**FIGURE 1:**
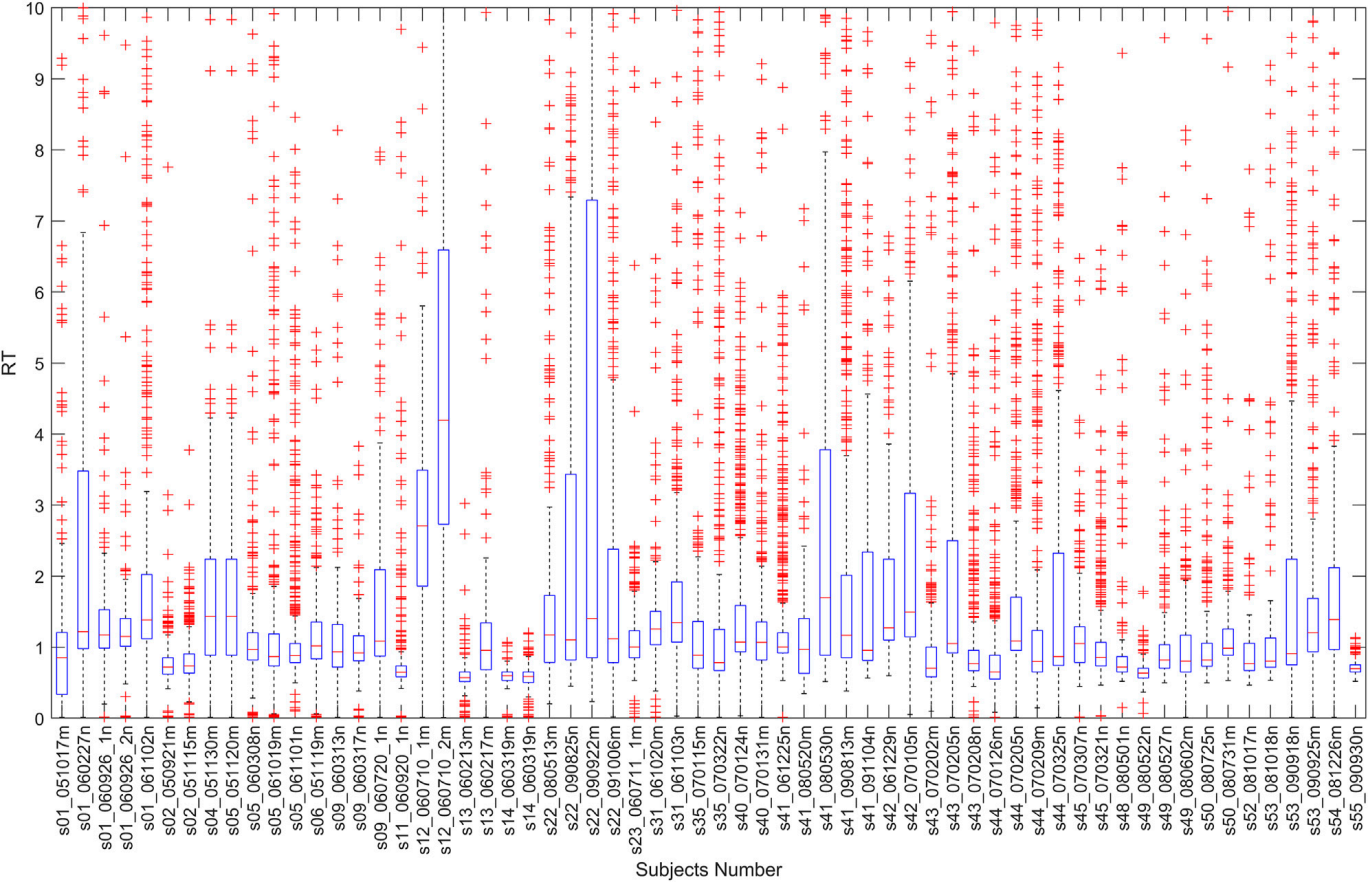
All trails’ reaction times.

**FIGURE 2:**
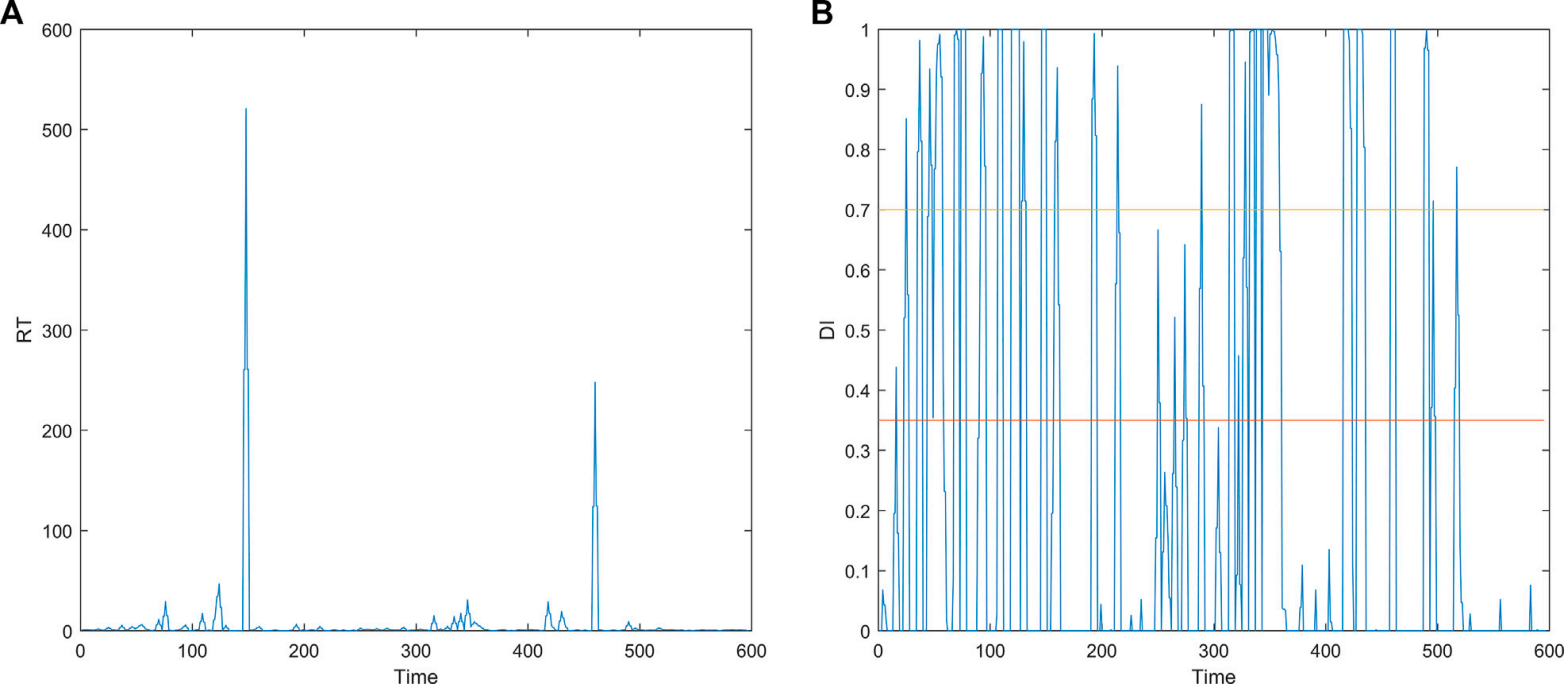
Curves before and after the transformation. **(A)** RT curve. **(B)** DI curve.

The RT is the time difference between the lane-departure event onset and the subject’s response onset. This study calculated all trails’ RT, and the boxplot of these is shown in Figure 1. As can be seen from Figure 1, each person’s RT is different, and it has a long-tail effect. Therefore, the RT τ is transformed into the drowsiness index (DI) (Yang et al., 2021a) by the following Equation 1 in this study. The RT has been proved to have a strong correlation with the drowsiness level, while the DI is positively correlated with the RT. Therefore, the DI can be used to indicate the drowsiness level. The curves before and after the transformation are shown in Figures 2A, B (take s01_051017m.set as an example). As can be seen from Figure 2B, the fatigue curve determined according to the performance observed during the execution of specific tasks fluctuates greatly.

$$DI=\max\left(0,\frac{1-e^{-\left(\tau -\tau_{0}\right)}}{1+e^{-\left(\tau -\tau_{0}\right)}}\right),$$ (1)

where $\tau_{0}$ was set to 1.

The transformation can normalize the RT to the interval [0,1] and overcome the long-tail effect. Like the SEED dataset, the data labels are defined in such a way that the EEG data are classified into three fatigue states (awake, fatigue, and drowsy) based on the DI index with two thresholds (0.35 and 0.7). Specifically, in the following study, this study uses “multi-channel_0” for awake, “multi-channel_1” for fatigue, and “multi-channel_2” for drowsy in the multi-channel dataset.

#### 2.2.3 Channel selection

**FIGURE 3:**
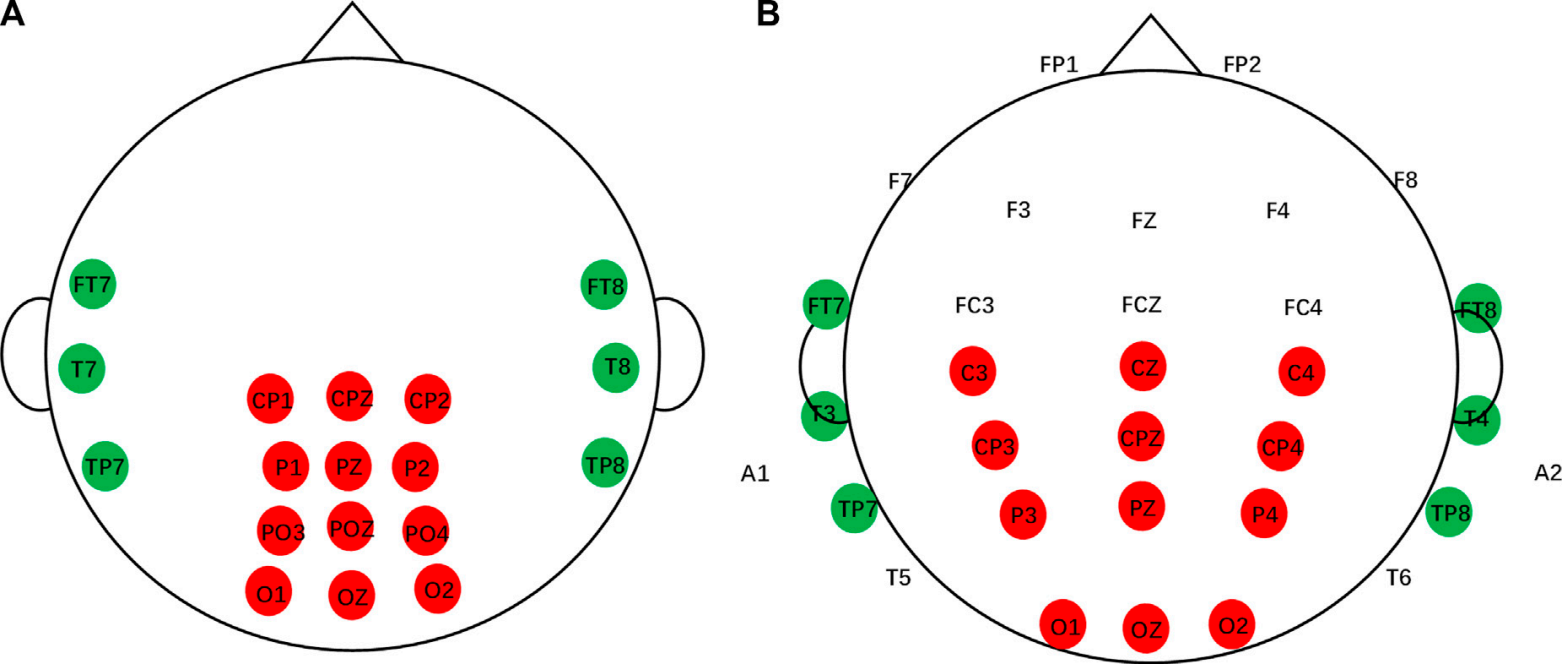
Channels of the SEED dataset and multi-channel dataset. **(A)** SEED dataset. **(B)** Multi-channel dataset.

For the EEG setup, the SEED dataset recorded 12-channel EEG signals from the posterior site (CP1, CPZ, CP2, P1, PZ, P2, PO3, POZ, PO4, O1, OZ, and O2) and 6-channel EEG signals from the temporal site (FT7, FT8, T7, T8, TP7, and TP8) according to the International 10–20 electrode system, as shown in Figure 3A.

The multi-channel dataset included 32 EEG signals and one signal for vehicle position. The first 32 signals were from the Fp1, Fp2, F7, F3, Fz, F4, F8, FT7, FC3, FCZ, FC4, FT8, T3, C3, Cz, C4, T4, TP7, CP3, CPz, CP4, TP8, A1, T5, P3, PZ, P4, T6, A2, O1, Oz, and O2 electrodes. Two electrodes (A1 and A2) were references placed on the mastoid bones. The next signal was used to describe the position of the simulated vehicle. This study compares the channels of the SEED dataset with those of the multi-channel dataset and selects the channels according to the one-to-one correspondence principle, as shown in Figure 3. In particular, if there are more datasets, channel selection should be based on the dataset with the lowest number of channels.

#### 2.2.4 EEG segmentation division

**FIGURE 4:**
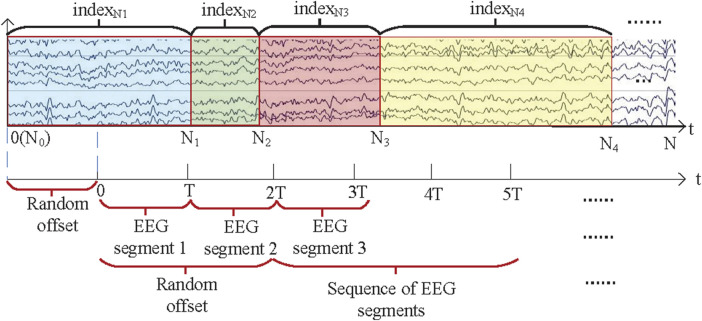
Random sampling method.

In our previous work, we found that the random sampling method can reduce overfitting. It is shown in Figure 4. The specific process is as follows. Assuming that the EEG sequence length is N and the sample length of the EEG segment is T, the EEG sequence of length N contains a number of EEG sub-sequence $\left[N_{i},N_{i+1}\right]$ , and each has its own index. For example, the EEG sub-sequence $\left[N_{0},N_{1}\right]$ corresponds to ${index}_{N_{1}}$ and the EEG sub-sequence $\left[N_{1},N_{2}\right]$ corresponds to ${index}_{N_{2}}$ , and so on. A random offset of $\left[0,T-1\right]$ will be set for the EEG sequence and EEG segments in each training iteration, which means that different EEG segments will be used in each iteration. The relationship between the EEG segment $\left[iT,\left(i+1\right)T\right]$ and the corresponding index ${index}_{\left[iT,\left(i+1\right)T\right]}$ is shown in Equation 2.

$${index}_{\left[iT,\left(i+1\right)T\right]}=\left\{\begin{array}{c} {index}_{N_{1},}\;if\;0<iT<N_{1},\\ \frac{\int_{iT}^{N_{1}}{index}_{N_{1}}d_{t}+\int_{N_{1}}^{\left(i+1\right)T}{index}_{N_{2}}d_{t}}{T},if\;N_{1}<iT<N_{2},\\ \frac{\int_{iT}^{N_{1}}{index}_{N_{1}}d_{t}+\int_{N_{1}}^{N_{2}}{index}_{N_{2}}d_{t}+\int_{N_{2}}^{\left(i+1\right)T}{index}_{N_{3}}d_{t}}{T},if\;N_{2}<iT<N_{3}.\\ \ldots ,\ldots \end{array}\right.$$ (2)

In this study, the EEG data on the SEED dataset were used as the independent variable and the PERCLOS was used as the dependent variable. In addition, the PERCLOS values provided in the SEED database were calculated every 8 s, so the PERCLOS values between two 8 s EEG segments were obtained by the aforementioned interpolation method. In the multi-channel dataset, EEG data were used as the independent variable and the DI as the dependent variable, and the DI values were obtained by the aforementioned interpolation method.

### 2.3 Proposed method

**FIGURE 5:**
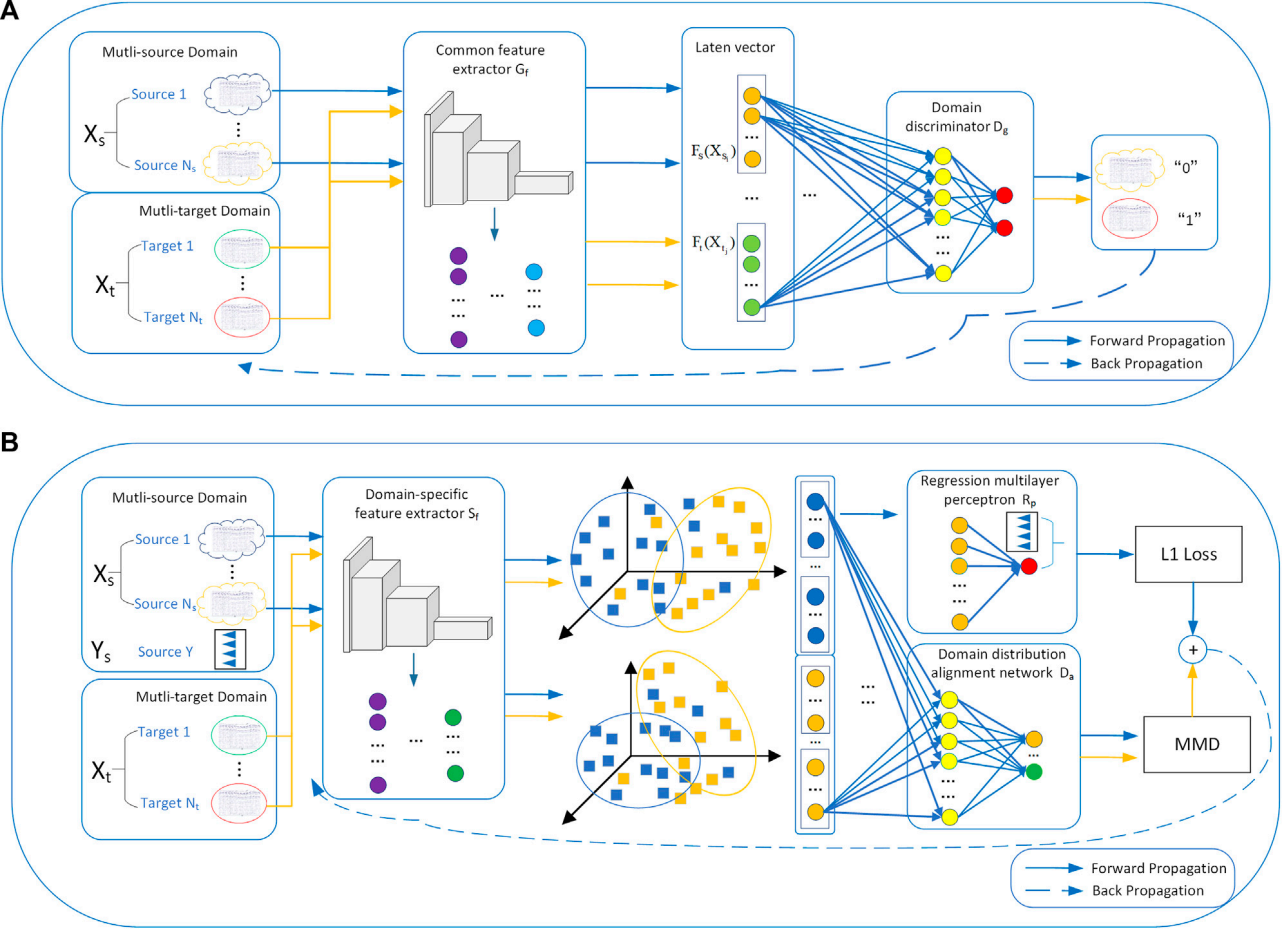
Regression method for EEG-based cross-dataset fatigue detection: **(A)** pre-training and **(B)** domain-specific adaptive step. In the pre-training step, the multi-source domain and multi-target domain data are input into $G_{f}$ and then fed into $D_{g}$ . In the domain-specific adaptive step, the specific features of the two domains are extracted by $S_{f}$ . Then, the distance MMD between the specific features is calculated by $D_{a}$ , and the fatigue index of the source domain is fitted by $R_{p}$ .

In order to get a fatigue detection model that can adapt to different tasks, this study proposes a regression method for EEG-based cross-dataset fatigue detection, as shown in Figure 5. In the pre-training step (Figure 5A), $X_{s}$ and $X_{t}$ are input into the common feature extractor $G_{f}$ to extract specific features ( $F_{s}\left(X_{s_{i}}\right)$ and $F_{t}\left(X_{t_{j}}\right)$ ) for different tasks. Then, the domain discriminator $D_{g}$ is used to determine whether these specific features come from $X_{s}$ (“0”) or $X_{t}$ (“1”). The cross-entropy loss is calculated and backpropagated to optimize the network. In this way, the specific features of different fatigue-induced tasks can be obtained.

In the domain-specific adaptive step (Figure 5B), this study proposes an EEG-based domain-adaptive fatigue detection network. In addition, it includes a domain-specific feature extractor $S_{f}$ , domain distribution alignment network $D_{a}$ , and regression multilayer perceptron $R_{p}$ . First, the specific features of $X_{s}$ and $X_{t}$ are extracted by $S_{f}$ . Then, the distance MMD (Chen et al., 2021) between the specific features is calculated by $D_{a}$ , and the fatigue index of the source domain is fitted by $R_{p}$ to calculate the mean squared error (MSE). Finally, the MMD and MSE were backpropagated to constantly update the network parameters and narrow the differences between features. The method can make the distribution domains of $D_{s}$ and $D_{t}$ more uniform, that is, $\lim_{t\to s}\left[P_{s}\left(X_{s}\right)=P_{t}\left(X_{t}\right)\right]$ and $\lim_{t\to s}\left[P_{s}\left(Y_{s}|X_{s}\right)=P_{t}\left(Y_{t}|X_{t}\right)\right]$ . Its aim is to extract the invariant features among domains and reveal the relationships between instances of the different datasets (Ye et al., 2022).

In terms of the design of a network structure, this study introduces the attention mechanism to extract the discriminative spatial representations and introduces the GRU to capture the relationship of EEG samples and the long-range information about EEG slices. The implementation processes of the proposed method are described in detail in Section 2.3.1 and Section 2.3.2.

#### 2.3.1 Design of a common feature extractor

In prior work, this study observed that the performance of shallower models more quickly saturated to lower performance levels, as compared to the deeper networks. If the shallow network depth is increased only, it is easy to cause overfitting and deteriorate the network performance. The residual network (ResNet) solves the problems of traditional convolutional neural network (CNN) degradation and gradient disappearance/explosion by adding jump connections.

Apart from these factors, this study investigates a different aspect of architectural design: attention. The significance of attention has been studied extensively in the literature (Mnih et al., 2014; Gregor et al., 2015). In addition, it plays an important role in deciding ‘where’ to focus, as shown in Chen et al. (2017). Woo et al. (2018) exploited CBAM, which is both channel-wise and spatial attention-based on efficient architecture. They integrated CBAM into ResNet and applied it to computer vision, and this method showed very good performance. Thus, the attention mechanism is very good at capturing spatial representations. However, there are few applications for fatigue detection (Wang H. et al., 2021).

**FIGURE 6:**
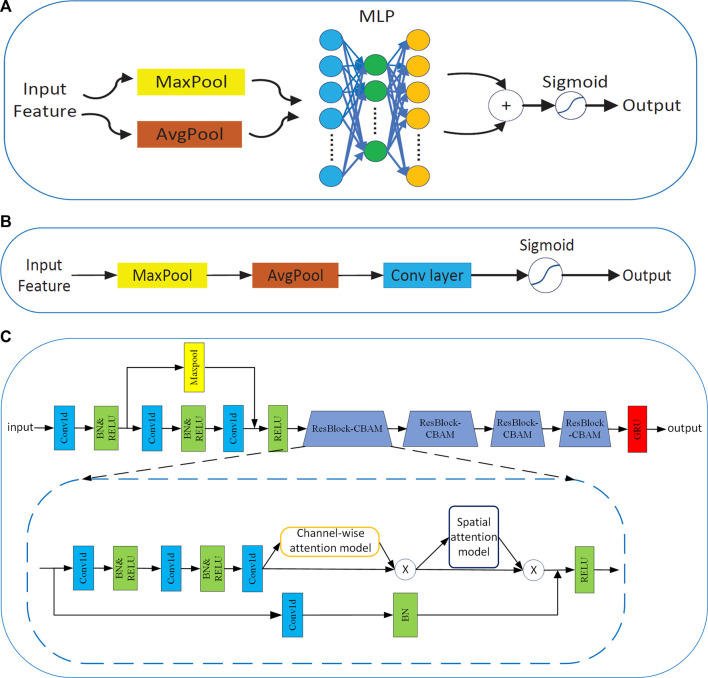
**(A)** Channel-wise attention model. **(B)** Spatial attention model. **(C)** Network structure of the common feature extractor.

In this study, ResNet50 was selected as the CNN to extract spatial local features of one-dimensional EEG samples. In addition, the CBAM network is integrated into ResNet50 by referring to the experiment of Woo et al. (2018). The channel-wise attention and spatial attention models are shown in Figures 6A, B. The method can adaptively estimate the importance of EEG channels without any prior information and effectively learn the discriminative spatial representations in EEG slices. In addition, several pioneering works mainly focus on the relationship of EEG samples or the connection between different channels, whereas few studies considered capturing the information about EEG slices. The GRU is added to capture the latent long-range temporal information on EEG signals. The network structure design of the common feature extractor $G_{f}$ is shown in Figure 6C.

#### 2.3.2 Domain-specific adaptive step

In the domain-specific adaptive step, this study builds the domain-specific feature extractor $S_{f}$ . The domain-specific feature extractor $S_{f}$ extracts multi-representations of $X_{s}$ and $X_{t}$ , that is, ${\left\{R_{X_{s_{i}}}\right\}}_{i}^{N_{s}}\;and\;{\left\{R_{X_{t_{j}}}\right\}}_{j}^{N_{t}}$ . $S_{f}$ maps them to a common subspace ∅ and preserves the key features of each domain and $S_{f}$ by using a $G_{f}$ -based shared feature extractor, i.e., $S_{f}=G_{f}$ , which is unlike the totally unshared architectures that require an extra network and increase the complexity of the model. Therefore, most domain-adaptive algorithms adopted this shared design (Eldele et al., 2022; He et al., 2022). To make these high-level features with different representations closer, this study employed MMD to estimate the distance between the domains in the latent space (Chen et al., 2021; Tao and Dan, 2021; Cao et al., 2022). In addition, it is calculated by the domain distribution alignment network $D_{a}$ . The final MMD is the sum of the MMD of each source domain expression and the corresponding target domain expression, as shown in Equation 3.

$$MMD\left({\left\{R_{X_{s_{i}}}\right\}}_{i}^{N_{s}},{\left\{R_{X_{t_{j}}}\right\}}_{j}^{N_{t}}\right)={\left\|\frac{1}{N_{s}}\sum_{i=1}^{N_{s}}\emptyset \left({\left\{R_{X_{s_{i}}}\right\}}_{i}\right)-\frac{1}{N_{t}}\sum_{j=1}^{N_{t}}\emptyset \left({\left\{R_{X_{t_{j}}}\right\}}_{j}\right)\right\|}^{2}.$$ (3)

Finally, the multi-representation vector ${\left\{R_{X_{s_{i}}}\right\}}_{i}^{N_{s}}$ is connected to a new vector and fed into the regression multilayer perceptron $R_{p}$ to predict the fatigue index. We use the MSE to calculate loss, as shown in Equation 4. The total loss function can be expressed as Equation 5.

$$MSE\left(\hat{Y},Y\right)=\frac{1}{N_{s}}\sum_{i=1}^{N_{s}}{\left(\hat{y_{i}}-y_{i}\right)}^{2},$$ (4)

$$L_{loss}=MSE\left(\hat{Y},Y\right)+\alpha \times MMD\left({\left\{R_{X_{s_{i}}}\right\}}_{i}^{N_{s}},{\left\{R_{X_{t_{j}}}\right\}}_{j}^{N_{t}}\right),$$ (5)

where α is the proportionality coefficient.

The training is based on Equation 5. Minimizing this formula is to minimize the MMD and MSE so that the distance between the source domain and the target domain can be as small as possible in different potential spaces, and the index prediction is as close as possible to the actual index.

In summary, the method proposed in this study follows the algorithm, as shown in Algorithm 1.

Algorithm 1: A regression method for EEG-based cross-dataset fatigue detection.

**Input:** The EEG data on the source domain and target domain, $X_{s}$ and $X_{t}$ .
The labels of source domain $Y_{s}$ , epoch N and batch size B, learning rate lr, parameters α ;
**Output:** prediction of target domain data, $\left\{\hat{Y}\right\}$ .
**Step 1: Pre-training**
Initialize the parameters of the model
1. **for** (epoch ← 1; epoch ≤ N; epoch ← epoch + 1) do
2. repeat
3. Sample source examples ${\left\{x_{s_{i}}\right\}}_{i=1}^{N_{s}}$ from $\left\{X_{s}\right\}$ ;
4. Sample target examples ${\left\{x_{t_{j}}\right\}}_{j=1}^{N_{t}}$ from $\left\{X_{t}\right\}$ ;
5. Sample labels “0” (source domain) and “1” (target domain);
5. Use $G_{f}$ to extract $F_{s}\left(X_{s_{i}}\right)$ and $F_{t}\left(X_{t_{j}}\right)$ ;
6. $F_{s}\left(X_{s_{i}}\right)$ and $F_{t}\left(X_{t_{j}}\right)$ are input into the $D_{g}$ to compute the cross-entropy loss;
7. Update the model by minimizing the cross-entropy loss;
8. **end for Step 2: Domain-specific adaptive**
Initialize domain-specific feature extractor $S_{f}$

9. **for** (epoch ← 1; epoch ≤ N; epoch ← epoch + 1) do
10. repeat
11. Sample source examples ${\left\{\left(x_{s_{i}},y_{s_{i}}\right)\right\}}_{i=1}^{N_{s}}$ from $\left\{X_{s},Y_{s}\right\}$ ;
12. Sample target examples ${\left\{x_{t_{j}}\right\}}_{j=1}^{N_{t}}$ from $\left\{X_{t}\right\}$ ;
13. Use $S_{f}$ to extract ${\left\{R_{X_{s_{i}}}\right\}}_{i}^{N_{s}}\;and\;{\left\{R_{X_{t_{j}}}\right\}}_{j}^{N_{t}}$ ;
14. ${\left\{R_{X_{s_{i}}}\right\}}_{i}^{N_{s}}\;and\;{\left\{R_{X_{t_{j}}}\right\}}_{j}^{N_{t}}$ are input into $D_{a}$ to compute $MMD\left({\left\{R_{X_{s_{i}}}\right\}}_{i}^{N_{s}},{\left\{R_{X_{t_{j}}}\right\}}_{j}^{N_{t}}\right)\;\left(3\right);$

15. Concat ${\left\{R_{X_{s_{i}}}\right\}}_{i}^{N_{s}}$ to regression multilayer perceptron $R_{p}$ to calculate the $MSE\left(\hat{Y},Y\right)$ (4);
16. The total loss is $L_{loss}$ (5);
17. Update the model by minimizing the total loss;
18. **end for** 19. Input $\left\{X_{t}\right\}$ into the updated model to predict;
20. **return** prediction of target domain data, $\left\{\hat{Y}\right\}$ .

## 3 Results

### 3.1 Dataset evaluation

In order to develop a model that can adapt to different fatigue tasks, we need to select a dataset with large information as the source domain and another as the target domain. Therefore, we need to reasonably judge the richness of each dataset and the information it contains.

**FIGURE 7:**
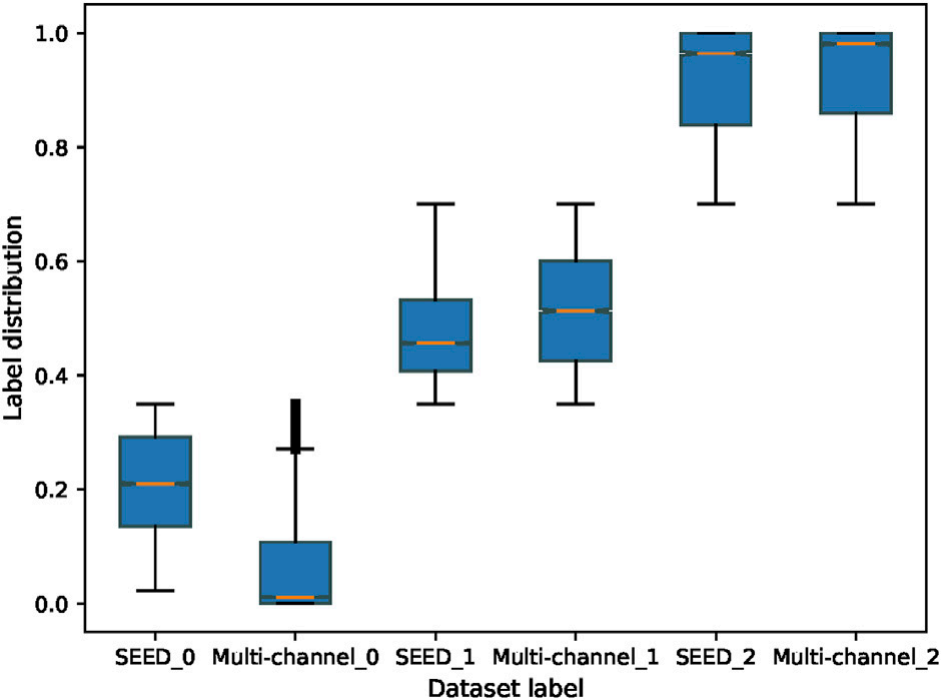
Fatigue index distribution of the SEED and multi-channel datasets. SEED_0, SEED_1, and SEED_2 indicate awake, fatigue, and drowsy states of the SEED dataset, respectively. Multi-channel_0, multi-channel_1, and multi-channel_2 indicate awake, fatigue, and drowsy states of the multi-channel dataset, respectively.

**FIGURE 8:**
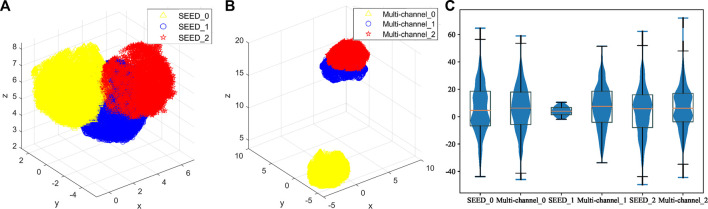
Distributions of the three states of the two datasets. **(A)** 3-D scatter plot of EEG samples of the SEED dataset. **(B)** 3-D scatter plot of EEG samples of the multi-channel dataset. Yellow color indicates the awake state of the two datasets, blue indicates the fatigue state, and red indicates the drowsy state in **(A)** and **(B)**. **(C)** Boxplot and violin plot of EEG samples of the two datasets. SEED_0, SEED_1, and SEED_2 indicate awake, fatigue, and drowsy states of the SEED dataset, respectively. Multi-channel_0, multi-channel_1, and multi-channel_2 indicate awake, fatigue, and drowsy states of the multi-channel dataset, respectively.

First, this study evaluates the distributions of two datasets. The SEED dataset and multi-channel dataset have different experimental tasks, so they may present different features. Therefore, the amount of information contained in each dataset should be evaluated comprehensively in order to select the appropriate source and target domains. Figure 7 shows the boxplot of the fatigue index distribution of the SEED and multi-channel datasets. Meanwhile, in order to see the distributions of the three states of the two datasets more directly, this study randomly picked out almost 256 EEG samples from the two datasets (each dataset has 128 samples) to visualize them with Uniform Manifold Approximation and Projection (UMAP) (Banville et al., 2021) *via* a 3-D scatter plot, as shown in Figures 8A, B. In addition, their boxplot and violin plot are displayed in Figure 8C.

It can be seen from Figure 7 that the fatigue index of the SEED dataset has a more centralized distribution with no outliers, while that of the multi-channel dataset has more outliers on “multi-channel_0.” Also, the multi-channel dataset is wider than the SEED dataset. It means that the changes of the multi-channel dataset are greater and scattered. In addition, it can be seen from Figure 8A that the SEED dataset has more transition features from awake to fatigue and from fatigue to drowsy states. However, the multi-channel dataset has less crossover (Figure 8B), which is not conducive to extract the transition features. We can observe from Figures 8A, C that the SEED dataset had a more concentrated EEG distribution and fewer outliers. Therefore, the SEED dataset is more suitable as the source domain. It is noteworthy that for different data, the range of the data after dimensionality reduction is different (Li et al., 2022). This study only shows the visualization effect of the proposed method here.

### 3.2 Experiment details

It should be noted although all the samples in the SEED and multi-channel datasets are labeled, the labels of the multi-channel dataset are used only for assessment but not for training. In the experiment, one sample size was 17*1024. In addition, the size of the GRU hidden layer is 64, and the number of hidden layers is 1. The optimizer uses a combination of the stochastic gradient descent and cosine gradually warm-up learning rate. In the cosine learning rate, every 100 epochs are half of the period of the cosine function, and the learning rate is 0.05 at most and 0.001 at least. In the gradually warm-up learning rate, the learning rate in the first 10 epochs is very small, and the learning rate starts to follow the change of the cosine learning rate from the epoch 11. The momentum in the optimizer is 0.9, and the weight decay rate is 0.001. α is 0.3. To avoid overfitting the source domain, this study also adds a dropout with a rate of 0.25 to the model. At each epoch, every 32 samples as a batch are used to train the network. The study used a single-layer multilayer perceptron (MLP) with one node as the regression multilayer perceptron and MLP with a 64-node domain distribution alignment network. All experiments are conducted in PyTorch libraries with an NVIDIA GeForce GTX 3060 GPU. All codes generated in this study are available at GitHub: https://github.com/yangyangyang-github/RMCDFD.

### 3.3 Performance evaluation

Different performance measurements, such as precision, recall, F1 score, accuracy, and root mean square error (RMSE), have been used to confirm the performance of the proposed method.

$$Precision=\frac{TP}{TP+FP},$$ (6)

$$Recall=\frac{TP}{TP+FN},$$ (7)

$$F1score=\frac{2*Precision*Recall}{Precision+Recall},$$ (8)

$$Accuracy=\frac{TP+TN}{TP+TN+FN+FP},$$ (9)

$$RMSE\left(Y,\hat{Y}\right)=\sqrt{\frac{1}{N}\sum_{i=1}^{N}{\left(y_{i}-{\hat{y}}_{i}\right)}^{2},}$$ (10)

where TP, TN, FP, and FN represent the number of true positive, true negative, false positive, and false negative values. $Y={\left(y_{1},y_{2},\ldots ,y_{N}\right)}^{T}$ is the true value, and $\hat{Y}={\left({\hat{y}}_{1},{\hat{y}}_{2},\ldots ,{\hat{y}}_{N}\right)}^{T}$ is the prediction.

### 3.4 Result

To validate the proposed method, this study compares the proposed method with the random value (33.33%). Likewise, we also compare the performance with that of other state-of-the-art domain adaptation methods, including transfer component analysis (TCA) (Pan et al., 2011), MIDA (Liu et al., 2020), InstanceEasyTL (Zeng et al., 2020), dynamic domain adaptation (DDA) (Li et al., 2022), and ADAST (Eldele et al., 2022). Meanwhile, in order to verify the effectiveness of source domain and target domain selection, this study compares the performance of all methods under two scenarios: 1) SEED → multi-channel and 2) multi-channel → SEED. These baselines are summarized as follows, and Table 1 shows the main results of five-fold cross-validation, which is averaged after 10 runs.

**TABLE 1:** Recall, precision, F1 score, accuracy, and RMSE performances.

	SEED → multi-channel					Multi-channel → SEED				
Method	Recall (%)	Precision (%)	F1 score (%)	Accuracy (%)	RMSE	Recall (%)	Precision (%)	F1 score (%)	Accuracy (%)	RMSE
**Proposed method**	**44.81**	**47.83**	**46.27**	**59.10**	**0.27**	**39.89**	39.26	**49.57**	**45.21**	**0.29**
TCA	34.26	36.52	35.35	42.38	0.34	33.56	34.21	33.88	34.61	0.31
MIDA	35.12	45.98	39.82	48.38	0.31	34.89	36.87	35.85	36.97	0.31
InstanceEasyTL	42.87	37.68	40.11	53.21	0.28	38.92	**41.79**	40.30	42.59	0.30
DDA	38.34	46.58	42.06	45.16	0.28	37.85	39.73	38.77	41.26	0.30
ADAST	40.21	39.64	39.92	50.36	0.27	39.46	38.45	38.95	38.95	0.30

The values in bold represent better results.

TCA: It seeks a projection to a latent subspace, where the projected source data and target data achieve a reduced MMD in a reproducing kernel Hilbert space, which measures the distance between the empirical means of two distributions.

MIDA: It uses the Hilbert–Schmidt independence criterion to evaluate the independence of potential subspaces and hopes that the maximum independence of subspaces can be achieved.

InstanceEasyTL: In order to match the different distribution of EEG signals from different subjects, it adopts a strategy of alignment with certain weights to align EEG samples collected from both source and target domains.

DDA: It introduces a dynamic training strategy where the model focuses on optimizing the global domain discrepancy in the early training steps and then gradually switches to the local subdomain discrepancy.

ADAST: It develops a mechanism to preserve the domain-specific features in both domains. In addition, it designs an iterative self-training strategy to improve the classification performance on the target domain *via* target domain pseudo labels.

Table 1 reports the recall, precision, F1 score, accuracy, and RMSE metrics of the proposed methods in two-domain transfer scenarios. It can be seen from Table 1 that the proposed method achieves a better result in the two-domain transfer scenarios. In the first scenario, the recall, precision, F1 score, and accuracy metrics achieved by the proposed method are 44.81%, 47.83%, 46.27%, and 59.10%, which significantly outperforms TCA by 10.55%, 11.31%, 10.92%, and 16.72%, respectively. In the second scenario also, the proposed method performs better than the others. In addition, the recall, precision, and F1 score metrics of the first scenario are about 5% higher than those of the second scenario, and the accuracy metric is 13.89% higher than that in the second scenario. Meanwhile, the RMSE in the first scenario was 0.02 higher than in the second scenario. Therefore, the comparison of the two scenarios in Table 1 verifies the effectiveness of the selection of the source and target domains. In the following experimental verification, the first scenario is taken as an example.

**FIGURE 9:**
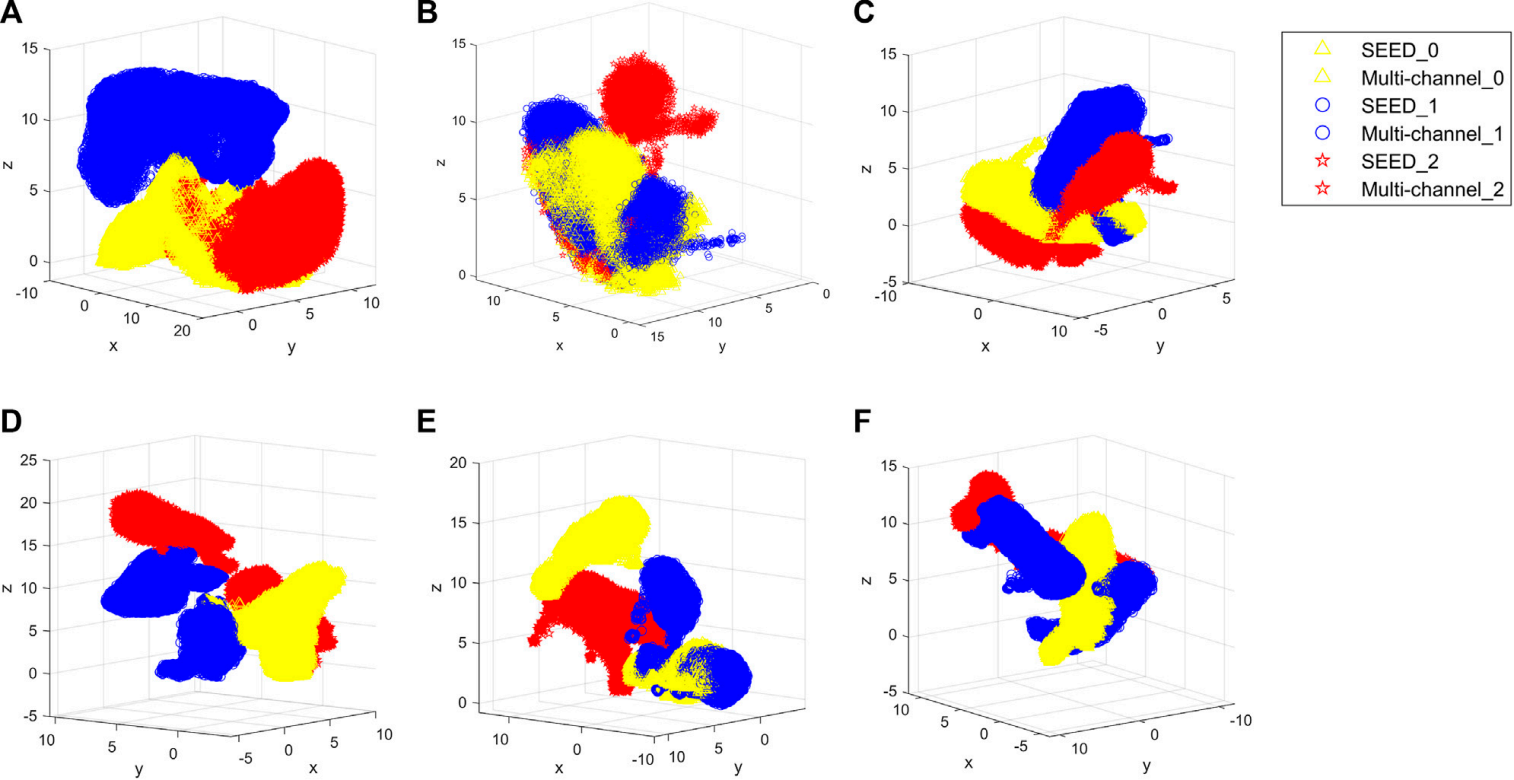
Visualized results of all methods. **(A)** Proposed method. **(B)** TCA. **(C)** MIDA. **(D)** InstanceEasyTL. **(E)** DDA. **(F)** ADAST. Yellow color indicates the awake state of the dataset, blue indicates the fatigue state, and red indicates the drowsy state. SEED_0, SEED_1, and SEED_2 indicate awake, fatigue, and drowsy states of the SEED dataset, respectively. Multi-channel_0, multi-channel_1, and multi-channel_2 indicate the awake, fatigue, and drowsy states of the multi-channel dataset, respectively.

In Table 1, the results illustrate the advantages of the proposed method over other methods. To make the comparison more intuitive, this study visualizes the results of all methods using UMAP (Banville et al., 2021), and they are shown in Figure 9.

### 3.5 Ablation experiment

In order to deeply understand the effect of attention, GRU, and pre-training, this study also compares the performance of the proposed method without attention (no attention), without GRU (no GRU), and without pre-training (no pre-training). It is also shown in Table 2.

**TABLE 2:** Cross-dataset fatigue detection results.

		Proposed method	No attention	No GRU	No pre-training
Accuracy (%)		59.10	49.56	44.45	46.74
Precision (%)	Awake	77.25	92.50	81.07	47.13
	Fatigue	17.46	20.30	17.81	46.67
	Drowsy	48.79	9.32	20.45	0
Recall (%)	Awake	73.71	57.76	52.44	23.57
	Fatigue	35.72	90.53	76.55	87.29
	Drowsy	25.00	0.24	1.13	0
F1 score	Awake	75.44	71.11	63.68	31.42
	Fatigue	23.45	33.17	28.89	60.82
	Drowsy	33.06	0.46	2.14	0
RMSE		0.27	0.27	0.35	0.27

As can be observed from Table 2, the proposed method’s accuracy is about 10% more than those of other methods. Specifically, the precision of the proposed method in the drowsy state is 40% higher than that of no pre-training, the recall is more than 20%, and the F1 score is more than 30%. It can also be seen from Table 2 that no pre-training method shows poor recognition performance in the drowsy state. The other three methods all have a pre-training part, and all of them perform better than the no pre-training model in terms of indicators of the drowsy state. Moreover, it is of note that although a certain module is removed, the proposed method is still better than the random value.

**FIGURE 10:**
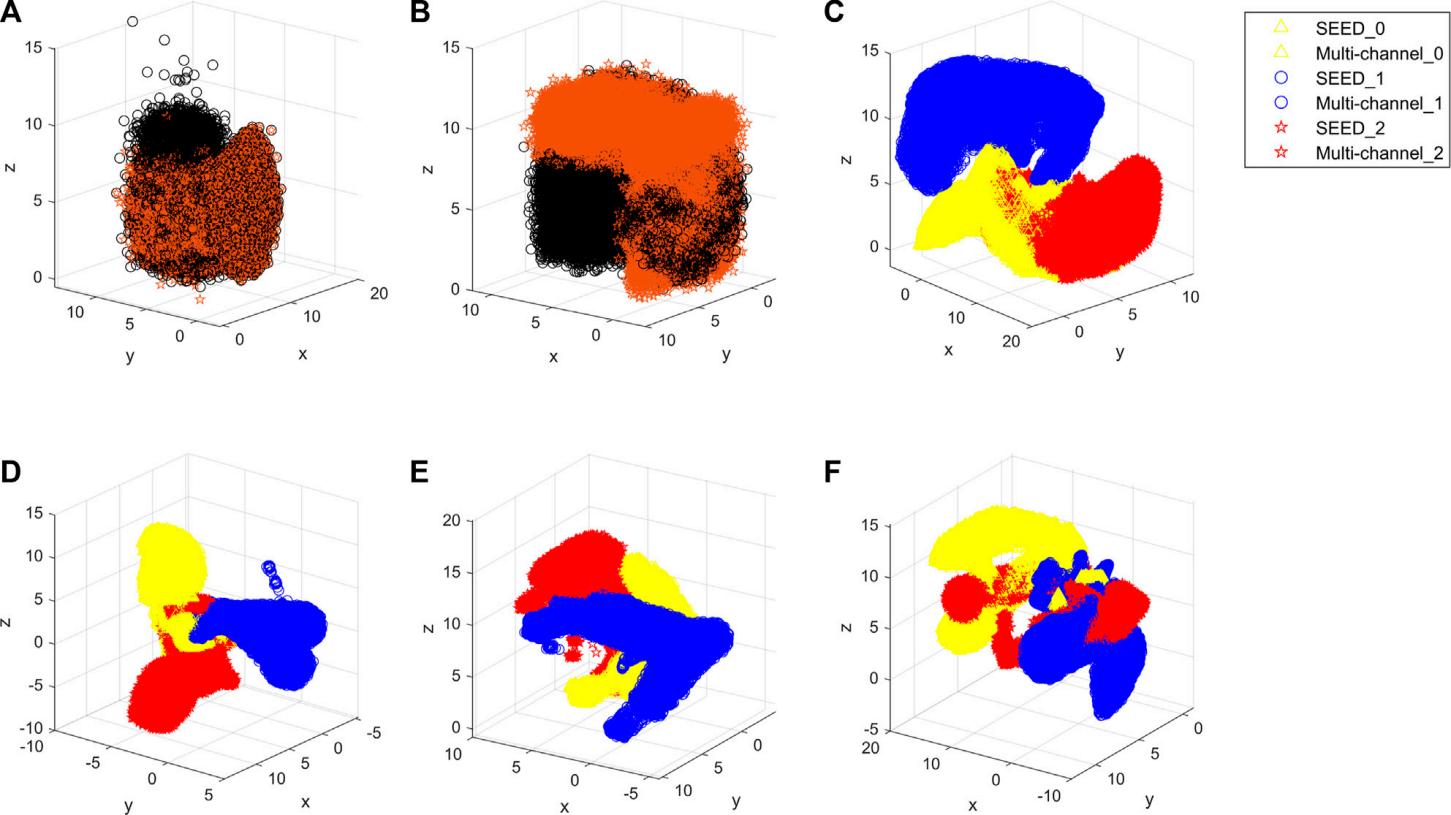
Outputs of the different stages. **(A)** Original EEG sample distribution of the two datasets. **(B)** Distribution after pre-training. Specifically, black indicates the SEED dataset, and red indicates the multi-channel dataset of **(A)** and **(B)**. **(C)** Distribution after the domain-specific adaptive step. **(D)** Distribution of no attention. **(E)** Distribution of no GRU. **(F)** Distribution of no pre-training. In **(C)**, **(D)**, **(E)**, and **(F)**, yellow indicates the awake state of the dataset, blue indicates the fatigue state, and red indicates the drowsy state. SEED_0, SEED_1, and SEED_2 indicate awake, fatigue, and drowsy states of the SEED dataset, respectively. Multi-channel_0, multi-channel_1, and multi-channel_2 indicate the awake, fatigue, and drowsy states of the multi-channel dataset, respectively.

In order to intuitively show how the proposed method reduces the distribution discrepancies between the domains, this study exhibits the outputs of the different stages *via* UMAP. Figures 10A–C show the distributions of original EEG samples, distributions after pre-training, and distributions after domain-specific adaptation, respectively. The distributions of the no attention, no GRU, and no pre-training are shown in Figures 10D–F, respectively. As can be seen from Figure 10B, the raw EEG data from the source and target domains are gathered into two groups by pre-training, which demonstrates the data distribution discrepancy between the source and target domains. Figure 10C proves that the proposed method reduces the difference at the domain level. It is obvious from Figures 10D–F that the three methods all have scattered data points that have not been aggregated, and the classification boundary is not obvious.

### 3.6 Effects of labeled data

**FIGURE 11:**
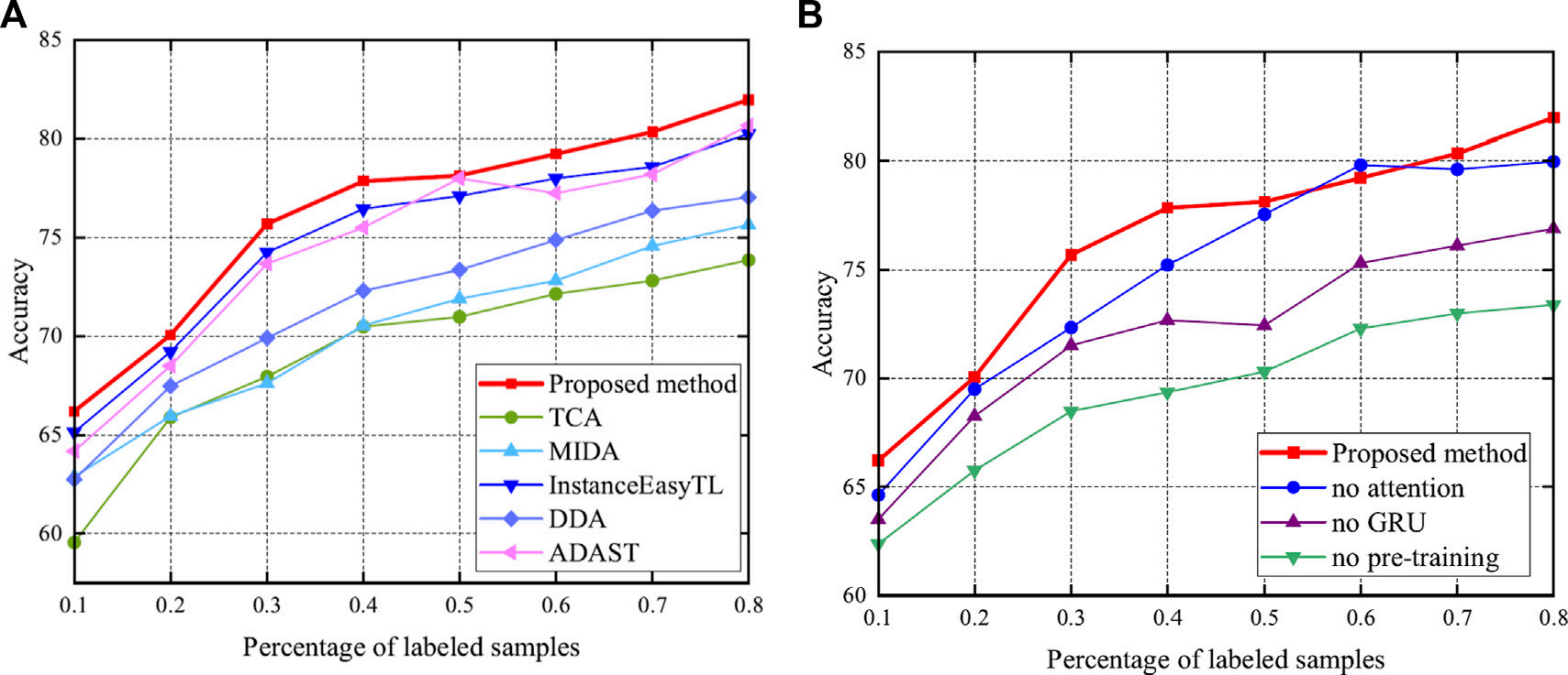
Accuracy after adding labels. Specifically, bold red indicates the accuracy of the proposed method. **(A)** Results compared with methods of Section 3.4. **(B)** Results compared with methods of Section 3.5.

**FIGURE 12:**
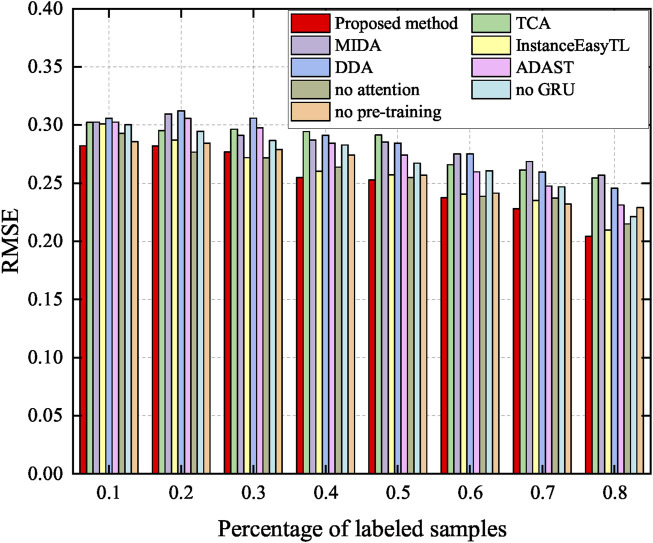
RMSE after adding labels. Specifically, red indicates the accuracy of the proposed method, which achieves better performance.

Utilization of a small amount of target labels can effectively improve accuracy (Li et al., 2022). Thus, this study deliberately investigates the relationship between the amount of target labels and the method performance. Here, 0.1, 0.2, 0.3, 0.4, 0.5, 0.6, 0.7, and 0.8 of the number of target labels are added to fine-tune the model. The performance of the proposed method is compared to the methods presented in Section 3.4 and Section 3.5. The accuracy and RMSE are shown in Figures 11, 12, respectively.

As shown in Figure 11, once the labeled data are added, the performance of the method significantly improves, as expected (Li et al., 2022). This emphasizes the importance of the labeled data. In particular, the proposed method shows excellent performance in a small number of labeled samples. Meanwhile, it can be seen from Figure 12 that the RMSE of the proposed method is kept at a low level, and with the increase of labeled samples, the RMSE of all methods decreases.

## 4 Discussion

In terms of fatigue detection methods, fatigue is mainly judged from facial expressions, physiological signals, and questionnaire surveys. The existing fatigue detection methods are almost within-subject and cross-subject fatigue detection. However, they need a large amount of EEG data for training, which is resource-consuming and impractical when faced with a new dataset. It is a worth exploring question to develop a model that can adapt to a variety of datasets.

Therefore, this study proposes a regression method for EEG-based cross-dataset fatigue detection. To validate the performance, this study compares the proposed method with that of other state-of-the-art domain adaptation methods. It can be seen from Table 1 that the proposed method outperforms all other methods, which does not need any labeled target data. Meanwhile, the comparison of the two scenarios in Table 1 verifies the validity of the selection of source and target domains, which means that the dataset with rich information is more suitable for the source domain. In addition, as can be seen from Figure 9A, the proposed method better aggregates each state, while other methods (Figures 9B–F) do not overlap the center of the same state.

For the proposed method, in the pre-training step, different domains should be mapped into the same space to distinguish samples of different datasets to extract specific features for different tasks. As can be seen from Table 2, the model without pre-training performs worse in the drowsy state than the model with pre-training. As can be seen from Figure 10F, the distribution of the model without pre-training is not concentrated in the drowsy state. Pre-trained models have a more aggregated distribution with fewer scattered data points. These validate the results of Table 2 and suggest that pre-training contributes to cross-dataset fatigue detection.

Then, the domain-specific adaptive step makes the multi-source domain and the multi-target domain closer, and the sample is highly aggregated. We can notice that this step can reduce the domain discrepancy at the domain level in the comparison between Figures 10B, C. It shows that it is effective to perform adaption alignment on top of specific features, which can avoid the occurrence of misalignment and learn fatigue-aware fine-grained transfer features (Li et al., 2022).

Since fatigue is a continuously changing sequence rather than several discrete states, the accuracies of no attention and no GRU are lower. We can see from Figures 10D, E that the conditional distribution of source and target domains using no attention and no GRU model matched, and aligned distributions are not well aggregated. As shown in the fatigue state in Figure 10D, there are still scattered points that have not been aggregated. In addition, fatigue and drowsy states are not concentrated in one area, so is the drowsy state in Figure 10E. This may be due to a lack of temporal and spatial information related to fatigue.

In addition, this study studies the effect of labeled samples on the results. We can observe from Figure 11 that the more labeled the data, the better the classification. This further validates the idea of supervised learning. However, considering a weak correlation between the target and source domains, blindly increasing the amount of source data does not improve the accuracy and causes computational burden (Wang Y. et al., 2021). It can also be seen from the results of Figure 11 that with the increase in the number of labeled samples, the performance does not increase monotonously. There is no denying that with the increase in the samples, the performance of the proposed method is clearly improved. At the same time, this also shows that if there are labeled samples in the actual target, then these samples should be used. We choose the unsupervised domain adaptation approach only when the target is completely unmarked.

Although this study proposes a regression method for EEG-based cross-dataset fatigue detection, there are still some limitations. Although the specific experimental design of the two datasets is different, they are all fatigue caused by driving tasks. The model may not perform well in the face of more fatigue datasets, such as those caused by sleep deprivation. Therefore, we will further study how to minimize the differences in fatigue caused by different tasks. In addition, the accuracy of the proposed method is only 59.10%. It is a little low, and part of the reason may be that the fatigue evaluation indexes are not necessarily 100% correct. The use of the behavioral index (RT in this study) to evaluate fatigue needs further development.

## 5 Conclusion

Since the cross-dataset fatigue detection model can extract general features of fatigue, it does not need to be retrained when facing new datasets. However, no one has studied this problem previously. Therefore, the study proposes a regression method for EEG-based cross-dataset fatigue detection, which mainly includes two steps: pre-training and the domain-specific adaptive step. In the pre-training step, this study designs a pretext task to distinguish data from the source or target domain. In this way, the specific features of different fatigue-induced tasks can be obtained. In the domain-specific adaptive step, this study proposes an EEG-based domain-adaptive fatigue detection network, including a domain-specific feature extractor, domain distribution alignment network, and regression multilayer perceptron. This step focuses on minimizing the data distribution difference between the source and target domains by using MMD. The accuracy and RMSE achieved by the proposed method are 59.10% and 0.27, respectively, which significantly outperforms state-of-the-art domain adaptation methods. In addition, this study also discusses the effect of labeled samples. When the number of labeled samples is 10% of the total number, the accuracy of the proposed model can reach 66.21%. The proposed method can be used for reference in the field of cross-dataset fatigue detection. In the future, we will investigate the EEG-based cross-dataset fatigue detection method due to different fatigue-induced tasks.

## Data Availability

The original contributions presented in the study are included in the article/Supplementary Material; further inquiries can be directed to the corresponding authors.
